# High Affinity Binders to EphA2 Isolated from Abdurin Scaffold Libraries; Characterization, Binding and Tumor Targeting

**DOI:** 10.1371/journal.pone.0135278

**Published:** 2015-08-27

**Authors:** Christopher Ullman, Pascale Mathonet, Arkadiusz Oleksy, Agata Diamandakis, Licia Tomei, Anna Demartis, Chiara Nardi, Sonia Sambucini, Antonino Missineo, Karen Alt, Christoph E. Hagemeyer, Matt Harris, Amos Hedt, Roland Weis, Kurt R. Gehlsen

**Affiliations:** 1 Isogenica, Ltd. Cambridge, United Kingdom; 2 IRBM Science Park, Rome, Italy; 3 Vascular Biotechnology, Baker IDI, Melbourne, Australia; 4 Clarity Pharmaceuticals, Ltd. Sydney, Australia; 5 VTU Technology, Grambach, Austria; 6 Research Corporation Technologies, Inc. Tucson, Arizona, United States of America; Centro Nacional de Biotecnologia—CSIC, SPAIN

## Abstract

Abdurins are a novel antibody-like scaffold derived from the engineering of a single isolated CH2 domain of human IgG. Previous studies established the prolonged serum half-life of Abdurins, the result of a retained FcRn binding motif. Here we present data on the construction of large, diverse, phage-display and cell-free DNA display libraries and the isolation of high affinity binders to the cancer target, membrane-bound ephrin receptor tyrosine kinase class A2 (EphA2). Antigen binding regions were created by designing combinatorial libraries into the structural loops and Abdurins were selected using phage display methods. Initial binders were reformatted into new maturation libraries and low nanomolar binders were isolated using cell-free DNA display, CIS display. Further characterization confirmed binding of the Abdurins to both human and murine EphA2 proteins and exclusively to cell lines that expressed EphA2, followed by rapid internalization. Two different EphA2 binders were labeled with ^64^Cu, using a bifunctional MeCOSar chelator, and administered to mice bearing tumors from transplanted human prostate cancer cells, followed by PET/CT imaging. The anti-EphA2 Abdurins localized in the tumors as early as 4 hours after injection and continued to accumulate up to 48 hours when the imaging was completed. These data demonstrate the ability to isolate high affinity binders from the engineered Abdurin scaffold, which retain a long serum half-life, and specifically target tumors in a xenograft model.

## Introduction

A number of antibody and non-antibody protein scaffolds are in development, which include the domain antibodies, V_HH_ domains from camelids, scFv, Fab, Abdurins, Affibodies, Adnectins, Centryns, and Darpins. These protein scaffolds are attractive due to their smaller size, their ease and low cost to manufacture, and flexibility for engineering molecules that can be multifunctional [1–9].

However, a major disadvantage of smaller protein scaffolds is rapid renal clearance resulting in a short circulating half-life of less than one hour [3, 6, 7, 10, 11, 12]. Several methods have been used to increase the serum half-life of small proteins, including fusion to albumin or the Fc domain, binding to free serum albumin, pegylation or fusion with half-life extension peptides [13, 14, 15, 16]. However, the use of these approaches may adversely affect the potential advantage of the scaffold’s small size and unfavorably alter the distribution and pharmacokinetics.

The Abdurin platform scaffold was developed to address the disadvantage of the shorter half-life for the smaller antibody-like scaffolds. Abdurins are isolated from the CH2 domain (heavy chain constant domain 2), are small (12.5kDa), and engineered to generate large libraries of binders to target molecules of interest. Importantly, Abdurins also retain a portion of the native FcRn binding motif which has been shown to bind FcRn protein in ELISA and *in vivo* [17, 18]. Pharmacokinetic studies in macaques, human FcRn transgenic mice and normal mice, demonstrated a circulating half-life in the 8–16 hour range [17]. Therefore, a smaller targeting protein retaining a prolonged half-life should provide measurable advantages over other small scaffold platforms, or even antibodies, in certain applications.

EphA2 is a member of the ephrin family of receptor tyrosine kinases [19]. The Eph receptors are involved in cellular proliferation, migration and angiogenesis. EphA2 has been shown to have no expression in most normal tissues and is highly expressed in several tumor types [20]. The level of EphA2 expression has been correlated to disease prognosis and, thus, is a promising target for cancer therapy [21]. Current approaches for targeting EphA2 include: antibodies, antibody-drug-conjugates, peptides, small molecules and vaccines. Several of these approaches report promising results in *in vitro* and *in vivo* tumor models. Both a small molecule multikinase inhibitor that targets EphA2 (dasatinib) and an antibody-drug-conjugate (MEDI-547) have completed phase 1 clinical studies [19, 21]. A comprehensive review on EphA2, its ligands, family members, and various targeting approaches has been published elsewhere [19].

We have developed large, diverse libraries of Abdurin binders and formatted these libraries for both phage and cell-free DNA display using CIS display [22, 23]. The Abdurin libraries were used to screen for specific binders to the extracellular domain of EphA2. A panel of high affinity Abdurin binders were isolated and characterized that recognize both murine and human EphA2 with low nM affinities. These binders are specific to the EphA2 receptor, readily internalized into cells and can specifically target EphA2-expressing tumors in a human prostate cancer xenograft model. Abdurins may offer certain advantages over monoclonal antibodies or other approaches for targeting tumors with imaging agents or delivering cytotoxic payloads due to their smaller size and prolonged half-life.

## Materials and Methods

### Chemicals and Materials

Synthetic genes were purchased from DNA2.0 (Menlo Park, California, USA). For plasmid isolation the PureYield Plasmid Miniprep System from Promega (Madison, Wisconsin, USA) was used. All DNA-modifying enzymes were obtained from Fermentas GmbH (Burlington, Ontario, Canada). Murine monoclonal antibody, SHM16, was purchased from EMD Millipore (Darmstadt, Germany). Chemicals were purchased if not stated otherwise from Becton, Dickinson and Company (Franklin Lakes, NJ, USA), Fresenius Kabi Austria (Graz, Austria) and Carl Roth (Karlsruhe, Germany).

For culturing *E*. *coli*, standard LB-medium containing 25 μg/ml Zeocin (Life Technologies, Paisley, United Kingdom) was used. For *P*. *pastoris*, YPhyD medium containing 10 g/L yeast extract, 20 g/L phytone peptone and 20 g/L glucose was used. For antibiotic selection in *Pichia* 100 μg/ml Zeocin (Eubio, Graz, Austria) was used. 15 g/L agar was added for plate media. 1% Buffered minimal media contained per liter: 200 ml 1 M sodium phosphate buffer (pH 6), 13.4 g yeast nitrogen base without amino acids, 0.0004 g biotin and 11 g glucose or 1% or 5% (v/v) methanol, respectively. All pre-cultures were prepared using YPhyD medium containing 20 g/L phytone peptone, 10 g/L Bacto-Yeast Extract and 20 g/L glucose. BSM medium contained per liter: CaSO_4_^.^2H_2_O 0.47 g, K_2_SO_4_ 9.1 g, KOH 2.07 g, MgSO_4_^.^7H_2_O 7.5 g, EDTA 0.6 g, H_3_PO_4_ (85%) 13.4 ml, Glycerol 40.0 g, NaCl 0.22 g and 4.35 ml PTM1. PTM1 Trace elements solution contained per liter: 0.2 g Biotin, 6.0 g CuSO_4_^.^5H_2_O, 0.09 g KI, 3.0 g MnSO_4_^.^H_2_O, 0.2 g Na_2_MoO_4_^.^2H_2_O, 0.02 g H_3_BO_3_, 0.5 g CoCl_2_, 42.2 g ZnSO_4_^.^7H2O, 65 g Fe(II)SO_4_^.^7H_2_O and 5 ml H_2_SO_4_. The fed-batch media were either 60% (w/w) glycerol or concentrated methanol and were supplemented with 12 ml/L PTM1 mineral salts solution. 2x TY medium contained per liter: 16g tryptone, 10g yeast extract, 5g NaCl.

### Abdurin library synthesis and construction

Four first-generation libraries were built by PCR using trinucleotide oligonucleotide primers synthesized by Ella Biotech, GmbH (Martinsried, Germany) and *Thermococcus kodakaraenis* KOD polymerase (EMD Millipore, Darmstadt, Germany). For each library, two PCRs were required to cover the whole gene. For each PCR, 100 pmol primers and 100 ng DNA template were used in 500 μl reactions. 25 cycles were performed under conditions according to the manufacturer’s instructions. 10 μg of each PCR fragment were digested with 100 units BsaI (New England Biolabs, Boston, Massachusetts, USA) in 250 μl reaction volume, and incubated 1hour at 37°C. Approximately 5.5 pmol of each PCR fragment was ligated in 350 μl reaction volume with 1400 units T4 DNA ligase (New England Biolabs, Boston, Massachusetts, USA). A PCR was then performed to add the restriction sites required for cloning into the phagemid vector and 800 ng template DNA was amplified with two primers in a 1 ml PCR reaction volume. 15 PCR cycles were performed. Correct library assembly was confirmed by DNA sequencing.

Abdurin libraries were subcloned in frame with pIII into pIFF6, a phagemid expression vector containing the pIII gene sequence of filamentous phage f1 for the expression of recombinant protein inserts at the amino-terminus of capsid protein 3. The pIFF6 vector (gift by Franco Felici, Universita degli Studi del Molise, Italy) is derived from the pC89 vector and contains the coding sequence for the capsid protein pIII in place of pVIII [24]. Briefly, 10 μg of DNA fragments for library #13, #15, #16 and #17 were digested with HF *EcoRI*/*BamHI* restriction enzymes (New England Biolabs, Boston, Massachusetts, USA), purified on Qiaquick columns (Qiagen, Hilden, Germany) and ligated into the *EcoRI*/*BamHI* dephosphorylated phagemid vector overnight at 16°C. Ligation products were phenol extracted, ethanol precipitated and transformed into TOP10F’ electro-competent cells. Transformations were plated on 2x TY/100 ug/ml Ampicillin/2% glucose (TYAG) in 24x24 cm Nunc Bioassay plates. Each library was ligated, transformed and plated separately, and for each a number of clones were collected equal to or 10-fold superior to the theoretical diversity. Phage rescue was performed by infecting cells at OD_600nm_ of 0.5 with M13K07 helper phage (New England Biolabs, Boston, Massachusetts, USA) at a multiplicity of infection (MOI) of approximately 10. After overnight growth, cells were pelleted and phages in the supernatant purified through a CsCl gradient. After dialysis and titration, libraries were frozen in 10% DMSO and stored at -80°C ready to use.

### Selection of Abdurins against EphA2

Selection against mEphA2 was carried out directly by coating the recombinant protein (EphA2-Fc chimera, R&D Systems, Minneapolis, Minnesota, USA) at 10 μg/well on a Nunc Maxisorp ELISA plate. Libraries #13, #15, #16 and #17 were pooled together and 10^11^ phages were pre-blocked in 5% milk/PBS 1x/Tween 0.05% (MPBST) for 1 hour at room temperature (RT). For selection, pre-blocked phages were incubated for 1 hour at RT with the protein in MPBST; after extensive washings with PBS 1x/Tween 0.05% (PBST), the bound phages were eluted with triethylamine (TEA) and used to infect 10 ml of a mid-log culture of TG1 *E*. *coli* cells. After 1 hour, a small aliquot was taken, diluted and plated out to titrate the number of selected clones. The remaining cells were centrifuged, resuspended into 1 ml of 2x TY and plated onto a 2x TY agar (TYAG) bioassay plate.

After overnight growth at 30°C, cells were harvested from the plate in 2x TY/16% glycerol and frozen at -80°C. For the next round of selection 50 ml of 2x TY were inoculated with 50 μl of selected clones, grown at 37°C up to an OD_600nm_ of 0.5 and superinfected with M13K07 at a MOI of 10. After 1 hour of incubation at 37°C, the medium was changed by centrifuging the cells and resuspending in 50 ml of 2x TY plus 100 μg/ml ampicillin and 25 μg/ml kanamycin (TYAK). Amplified phages were recovered after overnight growth at 37°C by centrifugation and 900 μl used for the next round of selection, following the procedure described above. During the screening either the phage pool from the different rounds of selection or the individual picked clones were tested in phage-ELISA.

### Affinity maturation of EphA2 binding Abdurins using CIS DNA display

To introduce diversity in loop BC, PCR was used to amplify the Abdurin binder genes as stated previously. The clones selected from the phage selection, E10 and A9 were used as the DNA template and the PCR fragments were digested and ligated together as stated above. The libraries were digested with *NotI*, whereas the gene expressing *repA* was digested with *Bsp120I*. 5.5 pmol of library DNA and *repA* were ligated together in 350 μl reaction, with 1400 units ligase. A PCR was finally performed with a long primer adding the *tac* promoter upstream of the libraries as described elsewhere [22, 23]. 1.8 μg of DNA were amplified in a 1 ml PCR reaction for 15 cycles. Correct library assembly was confirmed by DNA sequencing.

Selections were performed on mouse recombinant EphA2 (Fc chimera, R&D Systems, Minneapolis, Minnesota, USA) that was biotinylated in-house using EZ-Link Sulfo-NHS-LC-Biotin (Pierce, Rockford, Illinois, USA). Free biotin was removed with Zeba Desalt Spin column (Pierce, Rockford, Illinois, USA). Generally, *in vitro* transcription and translations (IVTT) were performed as previously described [22, 23]. 6 μg of DNA (3x10^12^ molecules) were expressed in 200 μl IVTT reaction mixture. After expression, the samples were diluted 5-fold in selection buffer containing 2% bovine serum albumin (BSA), in PBS. 83 nM biotinylated EphA2 was added to the blocked IVTT reaction mixture and incubated for 1 hour at RT while mixing on a rotary mixer. 100 μl streptavidin coated magnetic beads (M280, Invitrogen, Carlsbad, California, USA) were then added for 15 minutes, to pull down the binders. The beads were then removed from the selection buffer and washed four times with 1 ml PBS, 0.1% Tween-20 and once with PBS (30 seconds per wash). Bound DNA was eluted from the beads by incubation in 1x ThermoPol buffer (New England Biolabs, Boston, Massachusetts, USA), at 75°C for 10 minutes. The eluted material was added to a recovery PCR reaction, thereby producing input DNA for the next round of selection.

For subsequent rounds of expression, the resulting DNA from the preceding round was added to a fresh IVTT mixture and the selection process was repeated. The target concentration was decreased to 20 nM for the 2^nd^ round, 5 nM for the 3^rd^ round, 500 pM for the 4^th^ round and 50 pM for the 5^th^ round. Washing stringency was increased to five washes for 5 minutes for round two and then seven washes for 5 minutes for subsequent rounds.

The DNA outputs from the 3^rd^, 4^th^ and 5^th^ rounds were cloned in pET33b (EMD Millipore, Darmstadt, Germany) and transformed in Shuffle cells (New England Biolabs, Boston, Massachusetts, USA) for cytoplasmic expression. After induction overnight at 20°C, the bacterial cells were lysed using BugBuster Master Mix (EMD Millipore, Darmstadt, Germany), and the cytoplasmic fraction was diluted in blocking buffer (2% BSA in PBS).

ELISAs were performed to isolate Abdurins that bound EphA2. Nunc Maxisorp plates (Fisher Scientific, Loughborough, United Kingdom) were coated with 500 ng per well of streptavidin in PBS overnight at 4°C. The plates where then coated with 50 ng per well of biotinylated EphA2 in PBS for 30 minutes at RT. After blocking the plates with blocking buffer (2% BSA in PBS) for 1 hour, the diluted lysate cells were added to the plates and incubated for 1 hour at RT. Abdurin binders were detected using horseradish peroxidase-conjugated anti-FLAG M2 antibody (Sigma-Aldrich, St. Louis, MO, USA) and tetramethylbenzidine (TMB) peroxidase substrate followed by detection and reading at 450 nm in an absorbance plate reader. A selection of positive clones that showed a high signal for EphA2, were sequenced by Sanger sequencing (Cogenics Ltd, Takeley, United Kingdom) to obtain the Abdurin sequences.

### Abdurin expression in *E*. *coli* and ELISA analysis

Affinity matured clones were expressed in Shuffle cells, and induced for 22 hours at 20°C with 0.5 mM isopropyl β-D-1-thiogalactopyranoside (IPTG). The cells were lysed with BugBuster Master Mix and the Abdurin domains were then purified on a HisTrap HP column using the ÄKTA Protein Purification Systems (GE Healthcare, Uppsala, Sweden), followed by further purification by size exclusion chromatography through Superdex 75 10/300 GL (GE Healthcare, Uppsala, Sweden).

Proteins were coated on Nunc Maxisorp plates at the desired concentration in the range of 10 μg/ml in PBS overnight at 4°C then blocked with 3% Marvell/PBS for 1 hour, and incubated with pre-blocked phages in 3% MPBST for 1 hour at RT. After extensive washing with PBST, phage binding was detected by addition of anti-M13-HRP monoclonal antibody (GE Healthcare, Uppsala, Sweden) diluted 1:5000 in 3% MPBS incubated for an additional hour at RT. Development with TMB peroxidase substrate was followed by reading the absorbance at 450 nm.

To test binding of Abdurins to cells, the hEphA2 gene was transfected into HEK293-EBNATet cells under a stable doxycycline inducible promoter [25]. A stable hEphA2-expressing clone (HEK293-hEphA2) was isolated and induced by 24 hour treatment with 100 nM doxycycline (Sigma-Aldrich, St. Louis, Missouri, USA). Cells were fixed in 2% para-formaldehyde in PBS (PFA-PBS) for 20 minutes, washed with 1x PBS, blocked with 3% MPBS for 1 hour and incubated with 100 ul of phage supernatants for 2 hours. Binding was detected using anti-M13-HRP and development with TMB peroxidase substrate.

### Surface Plasmon resonance

Surface Plasmon resonance (SPR) interaction analyses were performed using a Biacore 3000 (GE Healthcare, Uppsala, Sweden). Human EphA2 (hEphA2) was immobilized on a CM5 chip by amine coupling according to the manufacturer’s instructions (Amine Coupling Kit, GE Healthcare, Uppsala, Sweden). Briefly, the surface of the sensor chip was activated for 7 minutes using a mixture of 0.1M N-hydroxy succinimide (NHS) and 0.4M N-ethyl-N'-[3-dimethylaminopropyl] carbodiimide (EDC) and, 1.2 μg/ml of hEphA2 in 10mM sodium acetate (pH 4.0) was injected for 7 minutes at 10μl/minute, and residual activated groups on the surface were blocked by a 7 minute injection of 1M ethanolamine (pH 8.5). The binding of Abdurins to the immobilized ligand was evaluated by a multi-cycle kinetic procedure in HBS-P running buffer (50 mM Hepes pH 7.4, 150 mM NaCl, 0.005% surfactant P-20) provided by the manufacturer (GE Healthcare, Uppsala, Sweden). The analyte was injected for 2.5 minutes at 40μl/minute until equilibrium and dissociation was monitored for 5 minutes. The sensor surface was regenerated with a 30 second of 0.05M NaOH, 0.5M NaCl, 0.005% SDS, following by extensive washing (6 minutes, 40μl/minute). The collected data and the kinetic parameters were evaluated with BiaEvaluation software v3.0. The experiments were repeated three times. All the reagents were purchased from GE Healthcare (Uppsala, Sweden).

### Abdurin binding on EphA2-transfected cells

Chinese Hamster Ovary K1 cells (CHOK1), (ATCC, Manassas, Virginia, USA) were transfected with transfection-ready hEphA2 cDNA (OriGene Technologies, Rockville, Maryland, USA) according to the manufacturer’s instructions. The CHOK1-EphA2 cells used in the binding assay were the progeny of a single cell clone, whose expression of EphA2 was verified by flow cytometry using a mouse anti-human EphA2 (R&D Systems, Minneapolis, Minnesota, USA). Abdurins were incubated at different concentrations (1nM to 7μM) with CHOK1-EphA2 cells. Binding was detected with mouse anti-HisTag-FITC antibody conjugate (AbD Serotec, Raleigh, North Carolina, USA). Cells were washed and resuspended in FACS buffer (PBS, 1% BSA, 0.05% NaN_3_) and 10,000 events were analyzed on the FACS Jazz instrument (BD Biosciences, Franklin Lakes, New Jersey, USA).

### Localization immunofluorescence analysis

Human prostate cancer (PC-3) cells (ATCC, Manassas, Virginia, USA) were seeded at a density of 18,000 cells/well in 96 well microtiter plates and incubated at 37°C/5% CO_2_ for 24 hours. The plates were then placed at 4°C for 10 minutes. After 10 minutes, the medium was removed and the Abdurins B6, B11 and WTCH2 (negative control) were diluted in pre-warmed fresh media and added to the wells at a concentration of 1 μg/well. A mouse anti-human EphA2 (EMD Millipore, Darmstadt, Germany) was used as a positive control. Plates were incubated at 4°C for 30 minutes, followed by incubation at 37^°^C for 60 minutes. Media was removed, the cells fixed with 2% PFA-PBS solution and incubated in the dark for 20 minutes at RT. After five washes in PBS, cells were permeabilized with 3% BSA-PBS 0.1% Triton X-100 for 1 hour at RT.

The primary antibodies for detecting Abdurins or the compartment markers in the co-localization experiments were diluted in 3% BSA-PBS 0.1% Triton X-100 (anti-FLAGM2 1:1000 Sigma-Aldrich, St. Louis, Missouri, USA: anti-LAMP1 1:500, Abcam, Cambridge United Kingdom; αEEA1 1:500, Abcam, Cambridge, United Kingdom) and incubated for 1 hour at RT. After 3 washings in PBS 0.1% Triton X-100 and a final wash in PBS, the secondary anti-Mouse-AF488 or the anti-Rabbit-AF594 antibodies (Molecular Probes-Life Technologies, Eugene, Oregon, USA) were diluted 1:3000 in 3% BSA-PBS 0.1% Triton X-100 and added and incubated for 1 hour at RT. Washings were repeated as previously described. For the nuclear staining, cells were incubated with 4’, 6-Diamidizo-2-phenylindolem dihydrochloride (DAPI, Molecular Probes-Life Technologies, Eugene, Oregon, USA) in PBS for 20 minutes in the dark. Images were acquired using INCell 2000 Analyzer (GE Healthcare, Uppsala, Sweden).

### Abdurin expression and purification in yeast

The synthetic genes for B6, B11, shWTCH2 (version of the wild-type CH2 domain that has the N-terminal seven amino acids truncated), and WTCH2 (unmodified wild-type CH2 domain) were cloned into the multiple cloning site of the Zeocin-resistance *E*. *coli/P*. *pastoris* shuttle vector pPpT4 [26] via *Xho*I/*Not*I sites, downstream of the wild type AOX1 promoter. Plasmids were linearized with *Bgl*II, ethanol-precipitated and desalted. Electro-competent *P*. *pastoris* CBS 7435 mut^S^ cells [26] were prepared and transformed with 2 μg of the *Bgl*II-linearized pPpT4 vector constructs according to [27]. Transformants were plated on YPD-Zeocin (100 μg/ml Zeocin) agar plates and grown at 28°C for 48 hours.

*P*. *pastoris* strains expressing Abdurins were cultivated in 96-deep well plates as previously described [28]. Pre-cultures of individual strains were grown in 50 and 200 ml YPhyD medium containing 20 g/L Bacto-Yeast Extract and 20g/l glucose in wide-necked, baffled shake flasks at 120 rpm at 28°C. Each bioreactor (Infors Multifors system, Infors AG, Bottmingen, Switzerland) containing 450 ml BSM-media (pH 5.0) was inoculated from the pre-culture to an OD600 of 2.0. During the batch phase *P*. *pastoris* was grown on glycerol (4%) at 28°C. At the beginning of the glycerol feeding phase the temperature was decreased to 24°C. For methanol-fed cultures, the fed-batch phase was started upon depletion of initial batch glycerol with 16 g/L/hour glycerol feed solution followed by methanol induction. The methanol-feed was set to 2 g/L/hour and was gradually increased within the next 70 hours to 6 g/L/hour. Likewise, the glycerol-feed was phased down during the first hour of methanol induction to 0 g/L/hour. Dissolved oxygen was set to 30% throughout the whole process. After 92 hours of methanol induction the bioreactor cultivations were stopped.

Microfluidic capillary electrophoresis using the LabChip GX II (Caliper LS, PerkinElmer, Waltham, Massachusetts, USA) was used to detect and quantify the Abdurin proteins. Briefly, several μl of culture supernatants or bioreactor samples (taken at different time-points throughout the process) were fluorescently labeled and analyzed according to protein size, using an electrophoretic system based on microfluidics. Internal standards enable approximate allocations to size in kDa and approximate concentrations of detected signals. Proteins were quantified by calibrating the integrated areas of the protein-specific peaks in the electropherograms to an external reference protein standard (Cytochrome c) of known concentration.

Chromatography was performed using an ÄKTA Avant 150 system with a HisPrep 16/10 FF Ni-NTA column (both GE Healthcare, Little Chalfont, United Kingdom). Buffers used were 20 mM NaPi, 500 mM NaCl, pH 7.4 (buffer A) and 500 mM Imidazole, 20 mM NaPi, 500 mM NaCl, pH 7.4 (buffer B). For each run, pH and conductivity of the samples were adjusted to the values of the loading buffer using NaOH and NaCl. Samples were filtered through a 0.2 μm filter prior to loading. The following protocol was established and applied for all three Abdurins: after equilibration at 0% buffer B for 2 column volumes (CV), bioreactor supernatant containing approximately 200 mg of each sample protein was loaded, followed by a wash step of 0.5 CV at 0% buffer B, a wash step of 3 CV at 8% buffer B (40 mM Imidazole) and an elution step of 3 CV at 60% buffer B (300 mM Imidazole). Samples were collected in 10 ml fractions. The column was washed at 100% buffer B (500 mM Imidazole) for 2 CV and re-equilibrated for the next run at 0% buffer B for 5 CV.

Cation exchange (CIEX) chromatography was used as a polishing step and for exchanging buffer to PBS, pH 7.4 (used as elution buffer in CIEX). Samples (pooled fractions) were diluted to conductivity < 8 mS/cm using MilliQ water and applied to a HiPrep 16/10 SP Sepharose FF column (GE Healthcare, Little Chalfont, United Kingdom). Buffer A used for column equilibration and washing was 20 mM Sodium Phosphate, pH 7.4. Buffer B used for step elution was PBS, pH 7.4 (Gibco/Thermo, Waltham, Massachusetts, USA). Eluate was collected in 14 ml fractions and pooled after analysis.

After purification via Ni-NTA- and CIEX-chromatography as well as concentration using ultra filtration (5 kDa cut-off; Vivaspin 20 devices, Sartorius, Göttingen, Germany), final samples were analyzed by microfluidic capillary electrophoresis using a comparison to a cytochrome c standard. The concentration was determined by spectrophotometric analysis at 280 nm, and the Endotoxin-content was measured by Limulus Amebocyte Lysate assay (Charles River, Wilmington, Massachusetts, USA).

### ^64^Cu-production

No-carrier-added ^64^Cu was produced with the IBA Nirta target (IBA, Louvain-la-Neuve, Belgium) by the ^64^Ni(p, n)^64^Cu reaction. The target was produced by direct electroplating of highly enriched ^64^Ni (> 99%, Isoflex, San Francisco, California, USA) onto an Ag disk (24 mm diameter x 1.0 mm thick disk). The plating cell was filled with ^64^Ni solution + NH_4_OH (total = 55 ml) and electroplating was carried out at 5.0 mA using a chopped saw tooth current for ~12–24 hours to give an average 20–50 μm ^64^Ni thickness.

Targets were irradiated using an IBA 18/9 cyclotron (IBA, Louvain-la-Neuve, Belgium) with an incident beam of 14.9 Mev (18 MeV degraded by 0.5 mm aluminium foil). The irradiated disk was then loaded into an IBA Pinctada module (IBA, Louvain-la-Neuve, Belgium) and the ^64^Ni plating dissolved in recirculating 3 ml 9–12 M HCl at 70°C using a peristaltic pump. Once dissolved, the solution was loaded onto an AG 1-X8 anion exchange cartridge for purification and the cartridge is washed with various concentrations of HCl to recover the ^64^Ni and elute impurities such as ^61^Co. ^64^Cu was recovered in ~2 ml of water. Typical production yields average 30 mCi end-of-synthesis (EOS) for a 4 hour irradiation at 35 μA with a ^64^Ni thickness of 25 μm, EOS 12 hours post end-of-bombardment (EOB) [29].

### Radiolabeling

The next generation ^64^Cu ligand 5-[(8-amino-3,6,10,13,16,19-hexaazabicyclo-[6.6.6]eico-1-yl)amino]-5-oxo-pentanoic acid hydrochloride (MeCOSar) was used. Prior to the animal imaging work, 100 μg each of MeCOSar-B6, MeCOSar-B11, MeCOSar-shWTCH_2_ (non-EphA2 binding negative control) and MeCOSar-IgG (positive control for EphA2 binding) conjugates were radiolabelled with 25 MBq ^64^Cu at RT for 30 minutes. A solution of 10 mM ethylenediaminetetraacetic acid (10 μl) was added and the reaction mixture incubated for 5 minutes. All samples were washed twice with PBS using spin columns (Millipore, Bayswater, Victoria, Australia, 2,000 molecular weight cut-off). Analysis/Quality control was performed using thin layer chromatography (silica gel 60, F254; EMD Millipore, Darmstadt, Germany) with 0.1 M citrate buffer (pH 5) as the mobile phase. Radiolabelled immunoconjugate (1.5 μl) was spotted at the origin, the strip was allowed to air-dry and developed. The strip was cut into three pieces and the radioactivity in each section counted using a gamma counter (Wizard single-detector gamma-counter, Perkin Elmer, Melbourne, Victoria, Australia).

### PC-3 mouse xenograft model

Human prostate cancer (PC-3) cells (ATCC, Manassas, Virginia, USA) were propagated by serial passage in RPMI 1640 medium, supplemented with penicillin (100 U/ml), streptomycin (100 mg/L), and 10% fetal calf serum (Biochrom/Millipore, Bayswater, Victoria, Australia) at 37°C in a humidified atmosphere of 5% CO2.

Six-week-old male NUDE mice (Animal Resources Center, Perth, Australia) were kept under sterile and standardized environmental conditions (20±1°C, 50±10% relative humidity, 12 hr light-dark cycle) and received autoclaved food, water and bedding. For tumor inoculation, 2 x10^6^ PC-3 cells in 100 μl PBS were mixed with 100 μl Matrigel (Collaborative Biomedical Products, Chicago, Illinois, USA) at 4°C and administered subcutaneously into the right shoulder of each animal. Growing tumors were palpated; diameters were measured by a caliper and recorded three times a week. This study was carried out in strict accordance with the recommendations in the Guide for the Care and Use of Laboratory Animals of the National Institutes of Health and approved by The Alfred Medical Research and Education Precinct Animal Ethics Committee (E/1232/2012/B).

### PET/CT Studies

Three animals in each cohort were injected via a lateral tail vein with ^64^CuMeCOSar-constructs (40–50 μg in 100 μl). PET scans were acquired 4, 24, and 48 hours after tracer injection using a NanoPET/CT preclinical imager (Mediso, Budapest, Hungary) with a 25 minute PET acquisition time, and coincidence relation of 1–3. Image reconstruction of the entire data set was performed with the following parameters- Ordered subset expectation-maximization (OSEM) with single-slice-rebinning (SSRB) 2D line of response (LOR), energy window, 400–600 keV; filter Ramlak cut off 1, number of iteration/subsets, 8/6. For the CT scans, an X-ray voltage of 45 kVp, an exposure time of 900 milliseconds and a pitch of 0.5 were used. A total of 240 projections over 360° of rotation were acquired. Projection data were rebinned by 1:4 and reconstructed using a RamLak filter, into a matrix having an isotropic voxel size of 96 μm. During imaging, the animals were anesthetized with a mixture of 3% isoflurane and 97% oxygen. The animal bed was heated. Image files of PET and CT scan were fused and analyzed using the analysis software InVivoScope version 2.0 (inviCRO, Boston, Massachusetts, USA). The radioactivity concentrations in the PET images were recalculated to provide data of Standardized Uptake Value (SUV) by dividing the radioactivity concentration (Bq/ml) by the injected radioactivity (Bq) per body weight (g). This study was carried out in strict accordance with the recommendations in the Guide for the Care and Use of Laboratory Animals of the National Institutes of Health and approved by The Alfred Medical Research and Education Precinct Animal Ethics Committee (E/1232/2012/B).

### Biodistribution studies

After the last scan, the animals were perfused with PBS and the tumor as well as the organs of interest, such as liver, kidney, lung, heart and muscle, were removed, wet-weight measured on a high precision balance and the level of radioactivity determined with an aliquot of injected solution as standard in the gamma counter (Perkin Elmer, Melbourne, Victoria, Australia) using an energy window between 450 and 650 keV. Results were expressed as % injected dose per gram (% ID/g) of tissue. The Alfred Medical Research and Education Precinct Animal Ethics Committee (E/1232/2012/B) approved all experiments involving animals. Three mice for each construct were sacrificed 48 hours after tracer injection.

### Statistical Analysis

All quantitative data is reported as mean +/- one standard deviation. Statistical analysis was performed using ANOVA followed by Tukey’s multiple comparison tests; with *p* < 0.05 considered statistically significant.

## Results

### Abdurin library construction and screening

The base Abdurin scaffold is derived from the human CH2 domain of IgG_1_. The sequence of the scaffold is found in Table 1 and the binding loops used for the library designs are highlighted. Four first generation Abdurin libraries were designed using three randomized loop structures previously described [2, 18] (Table 2). The libraries were built using trinucleotide primers, where each codon was replaced by an equimolar mix of codons encoding the selected amino acids: Glu, His, Tyr, Ala, Asp, Ser, Val, Arg, Gln, Gly, Pro and Leu.

**Table 1:** Amino acid sequence of the Abdurin scaffold.

GPSVFLFPPKPKDTLMISRTPEVTCVVVDV**SHED**PEVKFNWYVDGVEVHNAKTKPR**EEQYNST**YRVVSVLTVLHQDWLNGKEYKCKVSN**KALPA**PIEKTISKAK

**Table 2:** Loop library designs.

Library code	Loop BC	Loop DE	Loop FG	Theoretical diversity
**#13**	Ser267→Asp270			2x10e4
**#15**		Glu293→Thr299		4x10e7
**#16**			Lys326→Ala330	2.5x10e5
**#17**	Ser267→Asp270	Glu293→Thr299		8x10e11

### EphA2 binder selection

The DNA libraries were cloned into the phagemid and displayed on the surface of the filamentous phage M13 as p3 fusions. After electroporation in TOP10F’ cells, a number of independent clones sufficient to cover the entire diversity of the libraries were collected and approximately 100 clones from each library underwent DNA sequencing to confirm the correct reading frame and verify the expected sequence patterns. The libraries were individually rescued, purified and titrated, then used to select against immobilized EphA2. After three rounds of selection on coated murine EphA2-Fc fusion protein (mEphA2-Fc), the phage pool supernatants from the three rounds of selection were tested in ELISA on coated mEphA2-Fc to assess the enrichment for binding to the target. To identify the single positives, 192 clones were individually rescued and analyzed by ELISA. Ninety-five clones identified as positives were sequenced and were grouped into seven dominant variants based on amino acid sequence similarity (Table 3). Despite all three loops being engineered separately in individual libraries, the selected clones were all isolated from the loop DE library (library #15).

**Table 3:** Amino acid sequences of loop DE clones selected for EphA2 binding.

Clone	Loop DE Sequence	Copy Number
**D2**	QLDPLYG	2
**E10**	QYDPLYG	1
**H6**	RVDPLGG	1
**B8**	GYYALGG	58
**B11**	SYYALGG	1
**H3**	AYYALGG	17
**A5**	ERYVSYV	1

Two strong consensus motifs can be observed in the selected binders: the first, x-x-D-P-L-x-G in clones H6, E10 and D2 and the second, x-Y-Y-A-L-G-G in clones B8, B11 and H3. However, clone A5 did not share consensus with other sequences. The different sequence motifs may reflect binding to alternative epitopes on EphA2, however, this was not tested.

**Fig 1:**
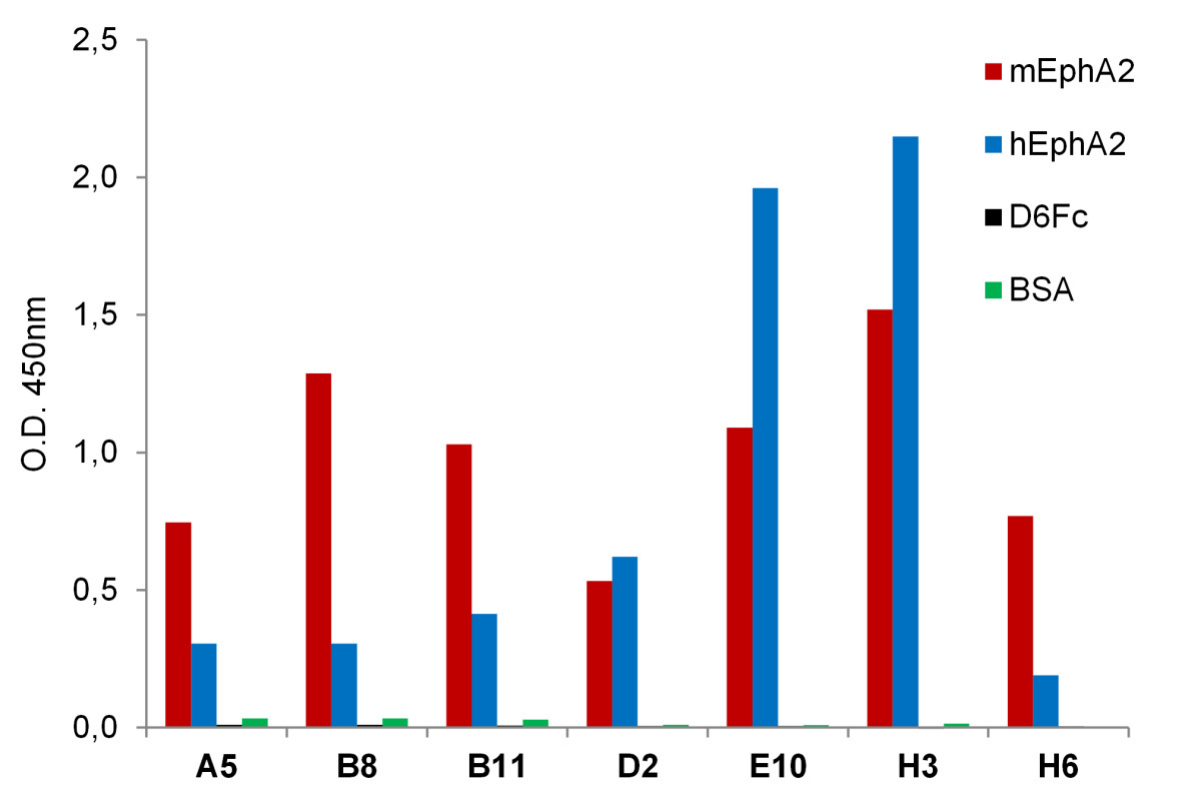
Phage ELISA on mEphA2-Fc, hEphA2, DR6-Fc and BSA. Phage supernatants of the seven clones with different loop DE sequences were tested by ELISA on plates coated with mEphA2-Fc, hEphA2, DR6-Fc and BSA to asses specificity of binding and crossreactivity.

**Fig 2:**
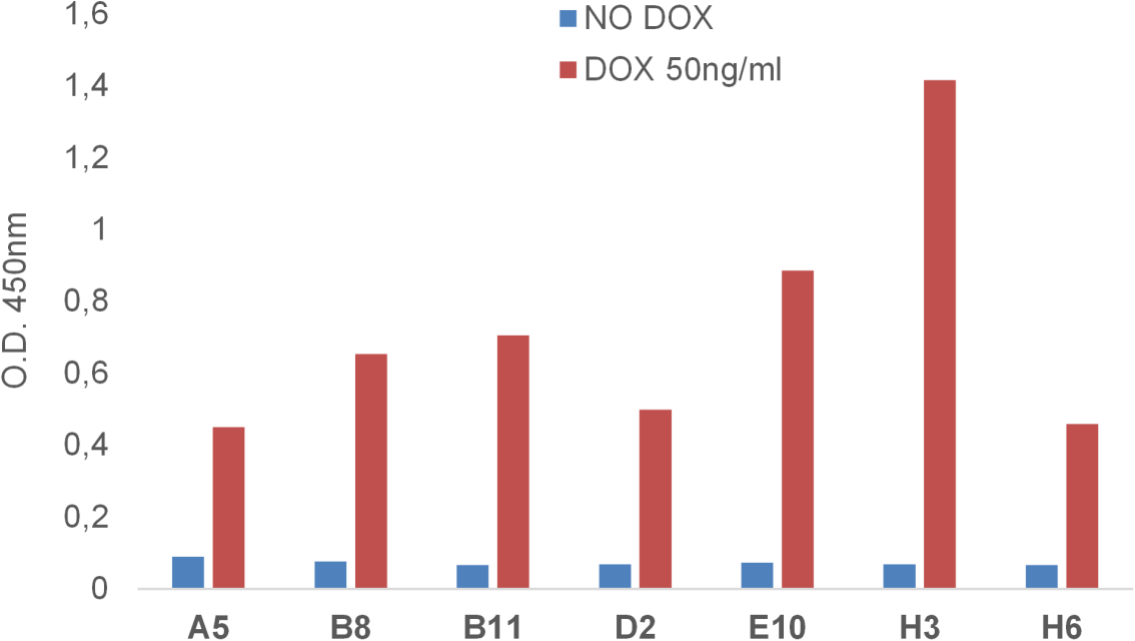
Phage ELISA on HEK293-hEphA2 transformed cells. Phage supernatants of the seven clones with different loop DE sequences were tested by cell-based ELISA on HEK293 cells expressing doxycycline-inducible hEphA2. All the Abdurin variants specifically recognize the hEphA2 receptor in its native conformation on cells. Assays were done in the absence or presence of doxycycline with concentrations of the phage ranging from 25 ng/ml to 100 ng/ml.

Binding capability and specificity of the seven unique clones was confirmed in ELISA on mEphA2-Fc, human EphA2 (hEphA2), death receptor-6-Fc fusion (DR6-Fc) and bovine serum albumin (BSA) (Fig 1). The isolated clones specifically bound both human and murine EphA2 recombinant proteins and did not recognize either the Fc domain linked to the mEphA2 antigen or unrelated proteins such as BSA or DR6. hEphA2 binding was also confirmed on stable HEK293-hEphA2 cells overexpressing the hEphA2 receptor in the presence of doxycycline. The Abdurin clones bound the human antigen when induced with doxycycline and no binding was observed in the absence of induction (Fig 2).

### EphA2 binder production and testing

EphA2 binders were expressed in *E*. *coli*, extracted and purified using a nickel affinity column followed by gel filtration on a Superdex HR75 16/60 column. Binding of E10, H3 and H6 variants to mEphA2 was analyzed by surface plasmon resonance. The *K*_*D*_ for mEphA2 binding ranged from 97 to 873 nM (Table 4).

**Table 4:** *K*_*D*_ measured with Biacore 3000 on mEphA2.

	mEphA2		
Binder	*K*_*D*_ (M)	*K*_*a*_ (1/Ms)	*K*_*d*_ (1/s)
**E10**	**9.7x10** ^**-8**^	7.91x10^4^	0.008
**H3**	**3.83x10** ^**-7**^	3.64x10^4^	0. 014
**H6**	**8.73x10** ^**-7**^	9.61x10^3^	0.008

### Affinity maturation of Abdurin binders

For the affinity maturation, clones E10 and H3 were chosen as representing two different consensus families and they showed higher expression and binding in the CIS display format. The affinity maturation libraries were built by introducing diversity into loop BC as it is in closer proximity to loop DE than is loop FG. Two different loop lengths were designed into loop BC, a smaller randomized loop where residues Ser267 to Asp270 were mutated, and a longer region of randomization comprising Val266 to Pro271. As before, the libraries were constructed using trinucleotide oligonucleotides incorporating the same twelve residues used in the primary libraries. Affinity maturation using pooled libraries was performed using CIS display on mEphA2-Fc, incorporating increased washing stringency and decreasing the target concentration in an attempt to select for the highest affinity binders. The output from the selection was cloned into a cytoplasmic expression vector, transformed in Shuffle cells and the expressed proteins screened by ELISA. From the ELISA, twenty-one mEphA2 binding clones were selected, sequenced and found to exclusively carry loop DE from the parental clone E10 (QYDPLYG). The loop BC binders were derived only from the longer length loop BC maturation library with no binders isolated from the shorter loop length library that met the binding affinity threshold of 200 nM or less.

The nine clones displaying the highest affinity were expressed and purified. The sequence of loop BC from the four best expressing clones demonstrated two distinct consensus sequences (Table 5). Clones D2 and B11 shared the motif Y-x-A-x-x-L and G7 and B6, P-x-L-x-x-D. The expression level was consistent with the motif and clones G7 and B6 were better expressed than D2 and B11 with D2 being the poorest expressing variant.

**Table 5:** Amino acid sequences for Loop BC variants.

Clone	Loop BC Sequences	Copy Number
**B11**	YRADYL	1
**D2**	YEAAAL	1
**B6**	PYLHDD	1
**G7**	PHLGVD	1

**Fig 3:**
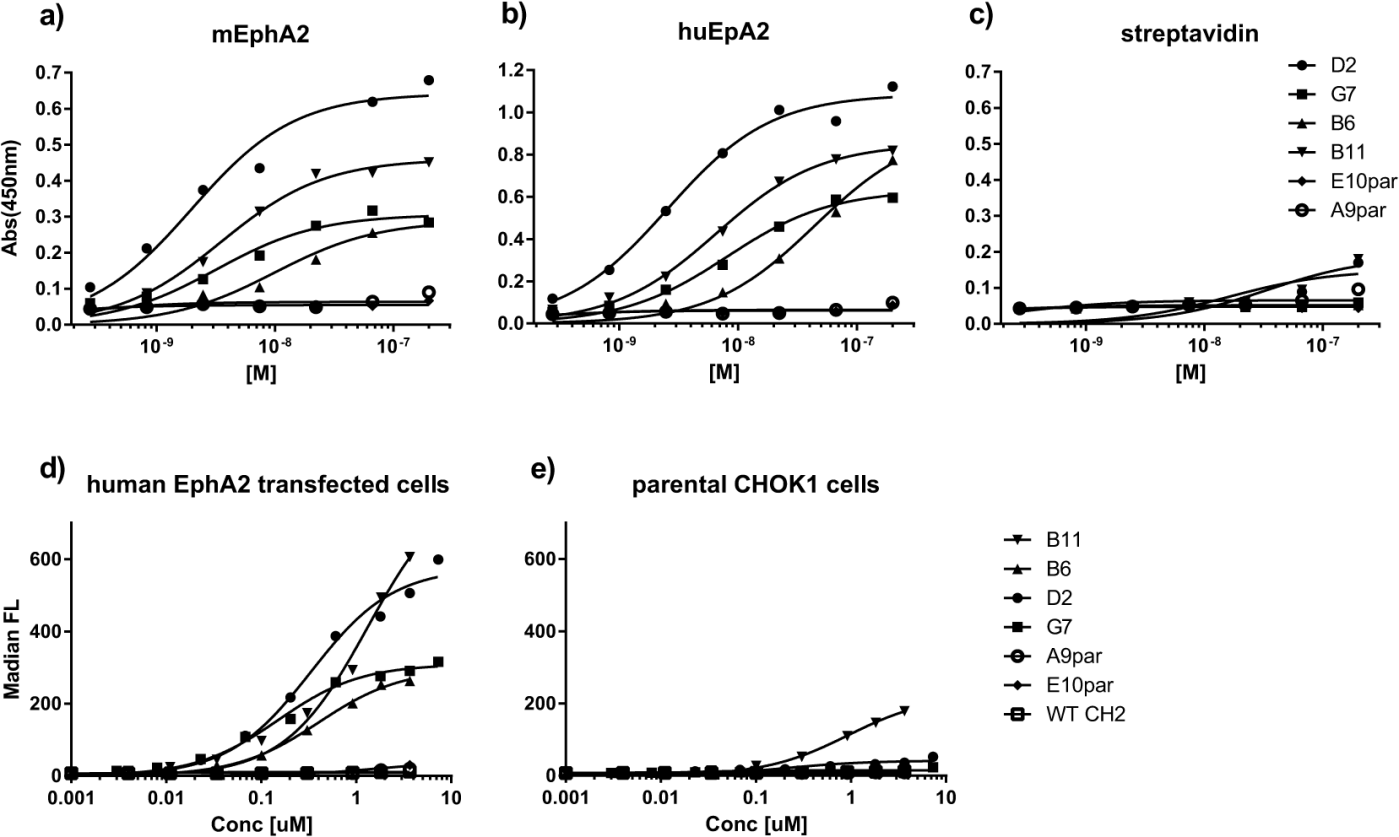
Abdurin binding assays. Affinity matured binders to EphA2, selected by CIS display, were tested in end-point titration ELISA assays in 96-well plates coated with hEphA2-Fc (a), mEphA2-Fc (b) or strepavidin (c). The purified matured Abdurin clones D2, G7, B6 and B11 were tested alongside the purified non-matured parental EphA2 binding clones selected by phage display, E10par and H3par, as detected by anti-FLAG M2-HRP conjugated antibody. Binding of Abdurin binders and the truncated wild-type human CH2 (shWTCH2) to CHO cells transfected with human EphA2 (d) or to non-transfected cells (e), was assessed by FACS analysis.

To determine the binding affinity of the clones, an end-point titration ELISA of the four anti-EphA2 Abdurins was performed against human and mouse EphA2 (Fig 3, Table 6). Binding of the parental clones, E10 and H3 could not be detected at concentrations tested, yet for the affinity matured clones low nanomolar values were calculated. Little difference in binding to either human or mouse EphA2 was observed, which is consistent with the fact that both proteins share 95% similarity [19].

**Table 6:** Affinity measurement for Abdurin binders to human and murine EphA2, determined by titration ELISA.

Clone	mEphA2		huEphA2	
	Affinity (M)	R^2^	Affinity (M)	R^2^
**B11**	9.89 10^−9^	0.89	4.43 10^−9^	0.98
**D2**	1.97 10^−9^	0.97	2.55 10^−9^	0.99
**B6**	3.50 10^−9^	0.98	6.54 10^−9^	0.99
**G7**	3.18 10^−9^	0.94	8.06 10^−9^	0.99
**E10par**	nd		nd	
**H3par**	nd		nd	

nd: no binding detected

**Fig 4:**
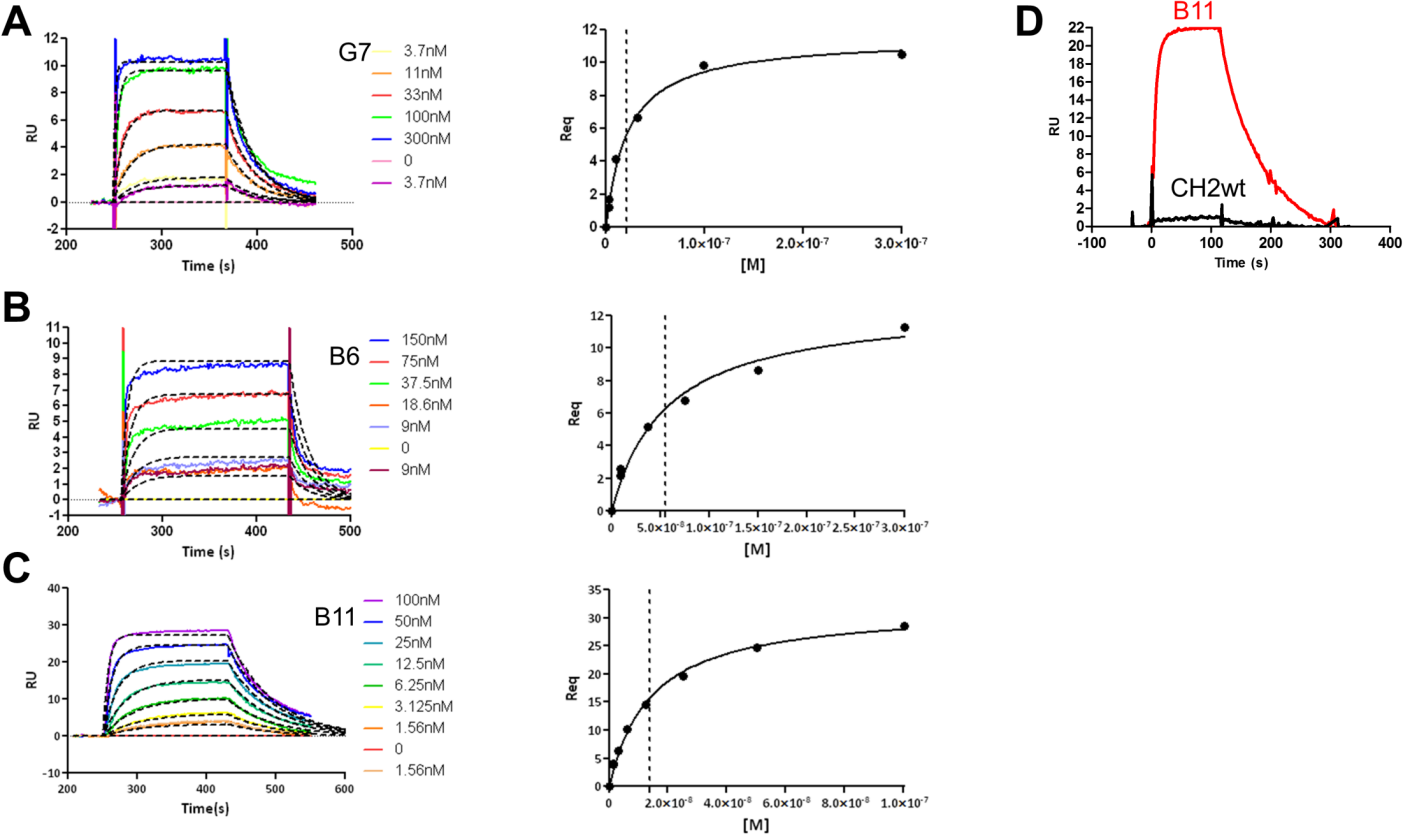
Surface Plasmon Resonance sensorgrams of Abdurin variants binding to hEphrinA2. hEph2A was immobilized on CM5 chip by amine coupling with a ligand density of 500 RU. Serial dilutions of analyte in HBS-P running buffer were injected over the ligand (2.5 min association time, 5 min dissociation time) followed by surface regeneration. Sensorgrams for: (A) G7 (3.7 to 300 nM), (B) B6 (9 to 150 nM), (C) B11 (1.56 to 100 nM), and (D) B11 (red sensorgram) or shWTCH2 (black sensorgram), were analyzed with BiaEvaluation software v 3.0 and kinetic parameters or affinity at the equilibrium were evaluated.

In order to measure the kinetic parameters of the interaction of the EphA2 binding variants and better compare their binding capabilities to hEphA2, binding kinetics for three of the most active variants (B11, B6 and G7) were analyzed using surface Plasmon resonance analysis. The hEphA2 protein was immobilized by amine coupling on a CM5 chip and increasing concentrations of the binders were injected followed by surface regeneration after each successive analyte injection. As shown in Fig 4, for all variants the sensorgrams obtained fitted well with a 1:1 binding model and kinetic *K*_*D*_ (*K*_*d*_ */K*_*a*_) was in full agreement with the affinity measured at equilibrium (Table 7). As a control, no binding to hEphA2 was observed with the shWTCH2 protein (Fig 4D). Binding to human FcRn (hFcRn) was also demonstrated for B6, B11 and shWTCH2 using surface Plasmon resonance analysis (Table 7).

**Table 7:** *K*_*D*_ measured using Biacore 3000.

	**hEphA2**			
**Binder**	***K***_***D* equilibrium**_ **(M)**	***K***_***D* kinetic**_ **(nM)**	***K***_***a***_ **(1/Ms)**	***K***_***d***_ **(1/s)**
**B11**	**1.4x10** ^**-8**^	**1.3 x10** ^**-8**^	1.21x10^6^	0.0158
**B6**	**5.6 x10** ^**-8**^	**6.9 x10** ^**-8**^	7.16x10^5^	0.0496
**G7**	**2.2 x10** ^**-8**^	**2.1 x10** ^**-8**^	1.80x10^6^	0.0375
	**hFcRn**			
**B6**	**3.8 x10** ^**-8**^	**1.0 x10** ^**-8**^	1.1x10^6^	0.011
**B11**	**2.6 x10** ^**-8**^	**1.2 x10** ^**-8**^	1. 1x10^6^	0.013
**shWTCH2**	**2.5 x10** ^**-8**^	**1.1 x10** ^**-8**^	1.5x10^6^	0.017

### Cell-based Assays

Chinese Hamster Ovary (CHO) cells were transfected with hEphA2 and the binding of the affinity matured Abdurins to cell-surface expressed hEphA2 was assessed by FACS (Fig 3). Decreasing concentrations of the B6, B11, D2 and G7 binders were tested and the affinities against the target expressed on cells were determined (Table 8). Of the four clones, B6 showed the highest affinity binding to the cells, with little binding to control CHO cells that did not express hEphA2, whereas B11 and to a lesser extent D2 generated a minor signal on non-transfected CHO cells (Fig 3). This background binding may be attributable to cross-reactivity with hamster EphA2 expressed in the immortalized ovarian cells and recognition of a different epitope than B6 and G7 (Chinese hamster EphA2 shares 92% and 94% amino acid similarity with human and mouse EphA2, respectively).

**Table 8:** Affinity measurement of binding to hEphA2 transfected cells.

Clone	hEphA2	
	Affinity (M)	R^2^
**B11**	4.03 10^−7^	0.99
**D2**	1.31 10^−6^	0.99
**B6**	1.51 10^−7^	0.99
**G7**	3.33 10^−7^	0.99
**E10par**	nd	
**H3par**	nd	
**shWTCH2**	nd	

nd: no binding detected.

E10par: the E10 parent loop DE binder

H3par: the H3 parent loop DE binder

shWTCH2: Control non-binding CH2D, truncated version

### Receptor binding and internalization

**Fig 5:**
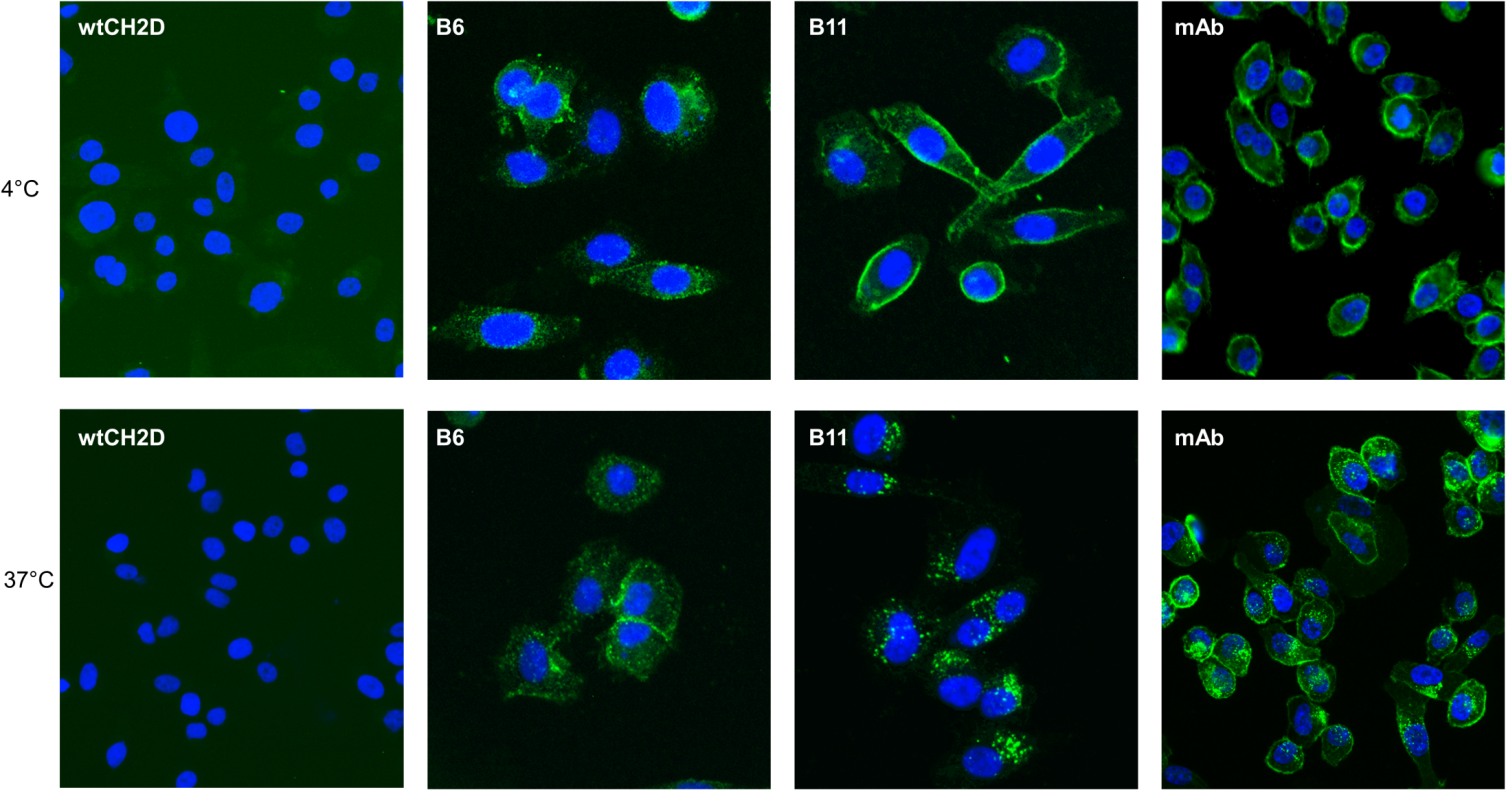
Temperature-dependent internalization of B11 into PC-3 cells. Top panels: PC-3 cells seeded in 96 well plates were incubated with 1 μg/well of B11, B6, negative control wild-type CH2 (wtCH2D) or 50 ng anti-EphA2 antibody (mAb) for 30 minutes at 4°C. Cells were then fixed in 2% PFA and binding of the Abdurins was monitored by an anti-FLAG antibody, followed by anti-mouse AF-488 secondary antibody. Binding of the anti-EphA2 antibody was monitored by anti-mouse AF-488. Bottom panels: Following incubation at 4°C, cells were moved to 37°C and further incubated for 60 minutes to allow internalization through the EphA2 receptor. Internalization of the EphA2-binders was monitored as above. Nuclei were stained with DAPI. Images were acquired using an IN Cell 2000.

**Fig 6:**
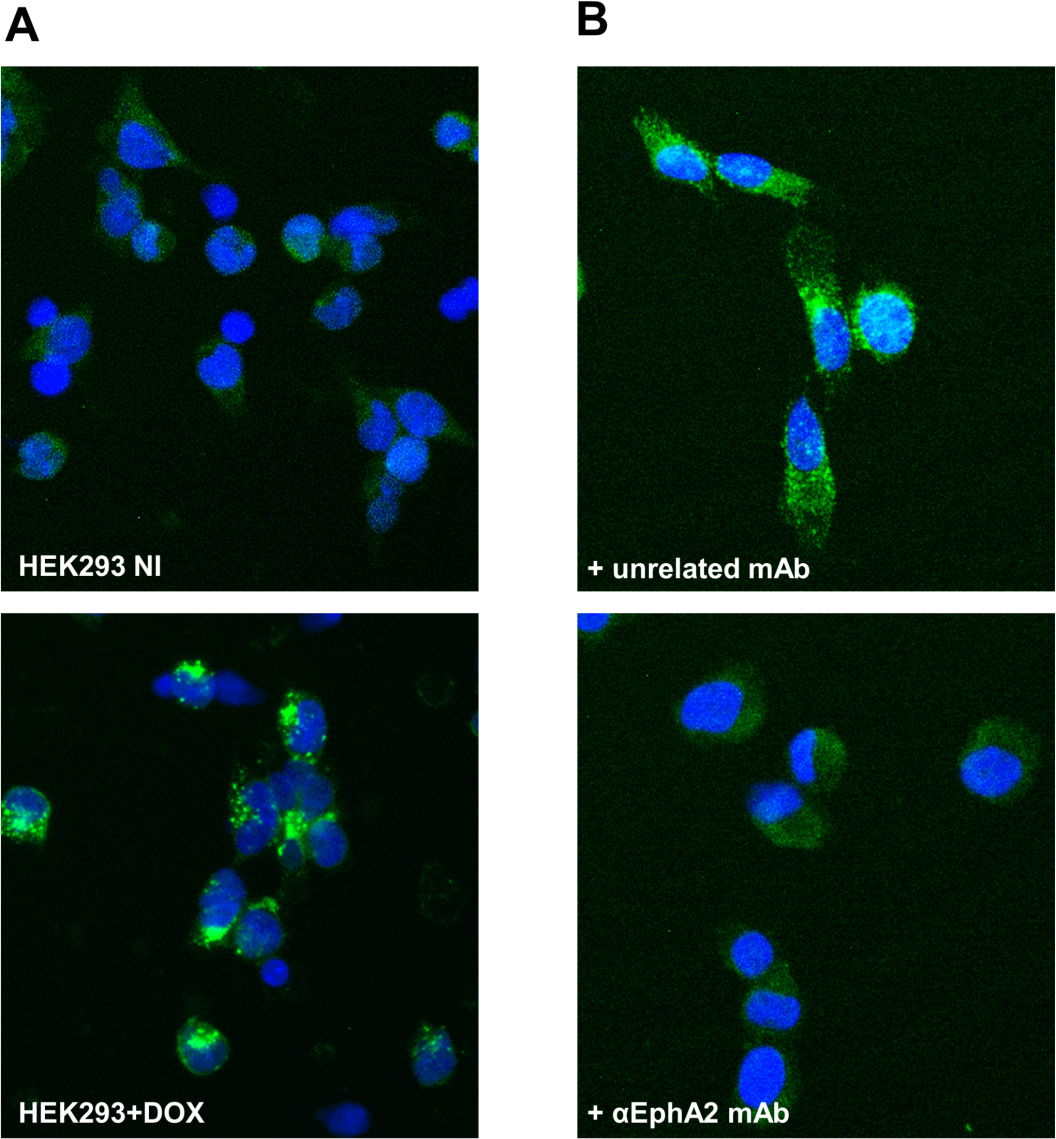
Specificity of B11 interaction and internalization. A) HEK293-hEphA2 cells were seeded in 96 well plates and either, not treated (top panel), or treated (bottom panel) with 100 nM doxycycline for 24 hours at 37°C to induce over-expression of the transfected EphA2 receptor. Cells were incubated with 1μg/well B11 for 30 minutes at 4°C after which time cells were fixed in 2% PFA. B11 binding was monitored by anti-FLAG antibody and anti-mouse AF488 secondary antibody. B) PC-3 cells seeded in 96 well plates were incubated with 1μg/well B11 and 10 μg/well of the anti-EphA2 monoclonal antibody (mAb) (molar ratio 1:1; bottom panel) or an unrelated mAb (top panel) for 30 minutes at 4°C followed by 1 hour incubation at 37°C to allow internalization. Cells were then fixed in 2% PFA and B11 binding monitored by anti-FLAG-AF488 antibody. Nuclei were stained with DAPI. Images were acquired at IN Cell 2000.

Binding and internalization of B6 and B11 through the endogenous EphA2 receptor expressed on PC-3 cells was evaluated by immunofluorescence experiments using a mouse anti-human EphA2 antibody as positive control and the wild-type human CH2 (wtCH2D) as a negative control. For the sake of clarity, both the wild-type CH2 (wtCH2D) and the N-terminal truncated (shortened) version of the wild-type CH2 (shWTCH2) have been used for various experiments and are interchangeable as control proteins. PC-3 cells were incubated with the various proteins at 4°C for 30 minutes to allow binding to the EphA2 receptor on the cell surface and to block internalization (Fig 5). After binding at 4°C, plates were moved to 37°C to allow internalization, and the cells imaged after 60 minutes. As shown in Fig 5, both B6 and B11 bound PC-3 cells (top panels) and internalized (bottom panels) similar to the anti-hEphA2 antibody. No fluorescent signal was observed on cells incubated with the wtCH2D. To further establish that the binding and internalization was specifically mediated by the hEphA2 receptor, internalization of B11 was completely blocked when cells were co-incubated with the anti-EphA2 antibody (Fig 6), but not with an unrelated antibody. This result confirmed the specific interaction of B11 to the hEphA2 receptor on the surface of cells and is supported by the binding of B11 to the HEK293-hEphA2 cells only upon induction with doxycycline. No binding was observed for B11on untreated HEK293-hEphA2 cells (Fig 6).

**Fig 7:**
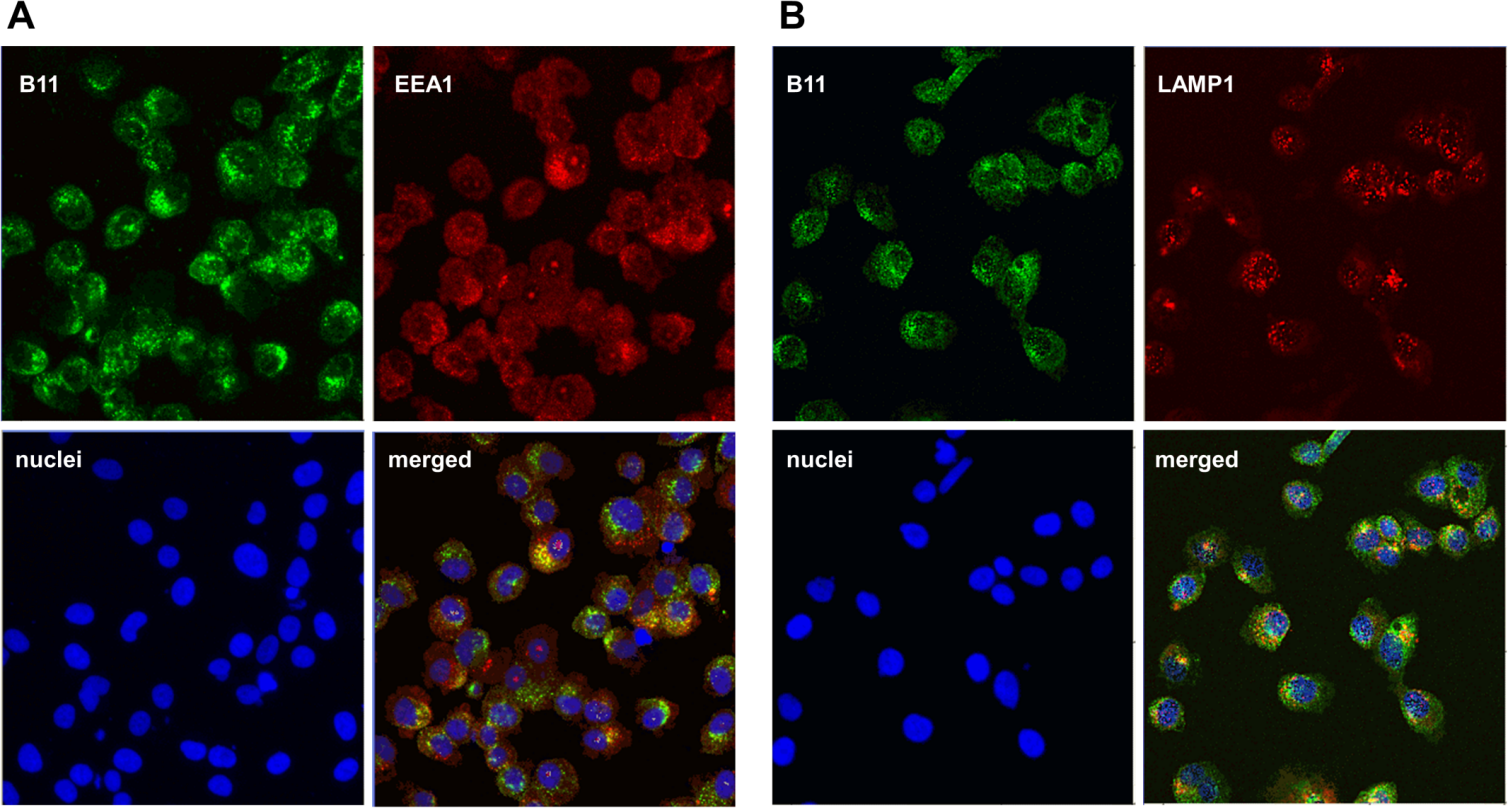
Localization of B11 in early endosomes and lysosomes. A) B11 internalization into early endosomes was followed by co-localization experiments using an anti-FLAG antibody (green) and the EEA1 protein, a marker of early endosome compartment, and imaged with the anti-EEA1 antibody (red). As shown in the merged images the two proteins co-localized (yellow) within the early endosome vesicles at 30 minutes. B) B11 trafficking into the lysosomes was followed by co-localization experiments using the anti-FLAG antibody (green) and anti-LAMP1 (red). Co-localization can be observed in the merged view (yellow) at 60 minutes.

Co-localization experiments to follow endosomal trafficking were performed using the anti-EEA1 antibody, an early endosome marker, in conjunction with an antibody to the FLAG-tag on B11. B11 appears to rapidly internalize, within 30 minutes, into early endosomes (Fig 7). Using an antibody to LAMP1 plus the anti-FLAG antibody, B11 begins to traffic into lysosomes at 60 minutes (Fig 7).

### Expression and purification of proteins for PET studies

Bioreactor cultivations under controlled conditions were performed using *P*. *pastoris* as the expression host. Titers of 900 mg/L (shWTCH2), 1,000 mg/L (B6) and 1,400 mg/L (B11) were obtained after a non-optimized standard 1 L bioreactor cultivation. Purities for all 3 target proteins were above 60% in the final filtrate and the filtrates were affinity-purified by immobilized metal ion affinity chromatography, polished using cation exchange chromatography, and the final protein concentrated by ultra filtration. Purified proteins were tested for hEphA2 and hFcRn binding using ELISA and surface Plasmon resonance assays and tested on EphA2-expressing cells lines, PC-3 and MBA-245 cells.

### PET/CT Imaging

**Fig 8:**
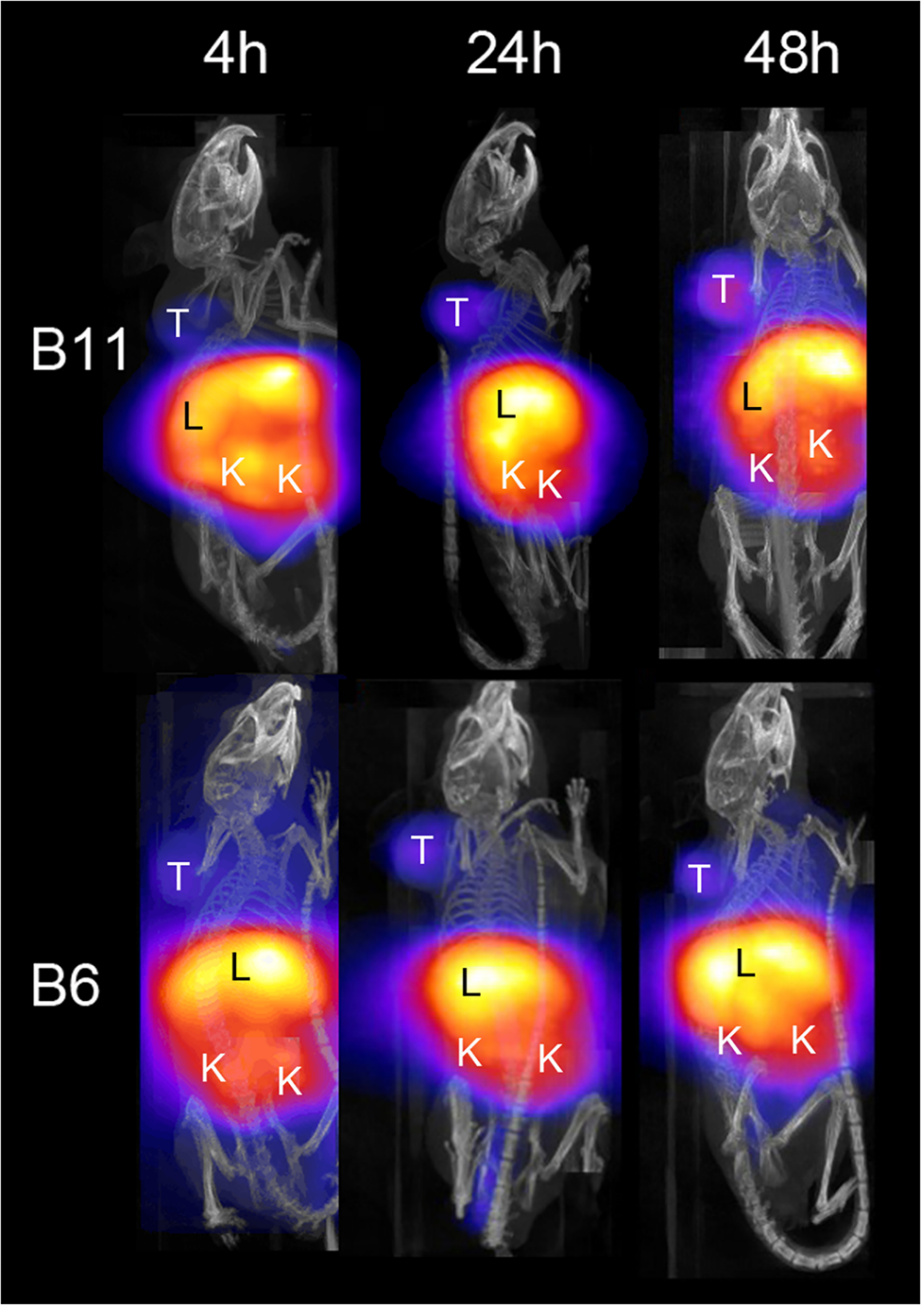
PET/CT imaging of mice bearing PC-3-tumors. Animals were imaged at 4 hours, 24 hours and 48 hours after injection of the ^64^Cu-MeCOSar radiotracer for B6 and B11. Shown are the maximum-intensity projections. The color scale for the PET image data shows radiotracer uptake with white corresponding to the highest activity and blue to the lowest activity. T: tumor; K: kidney; L: liver.

**Fig 9:**
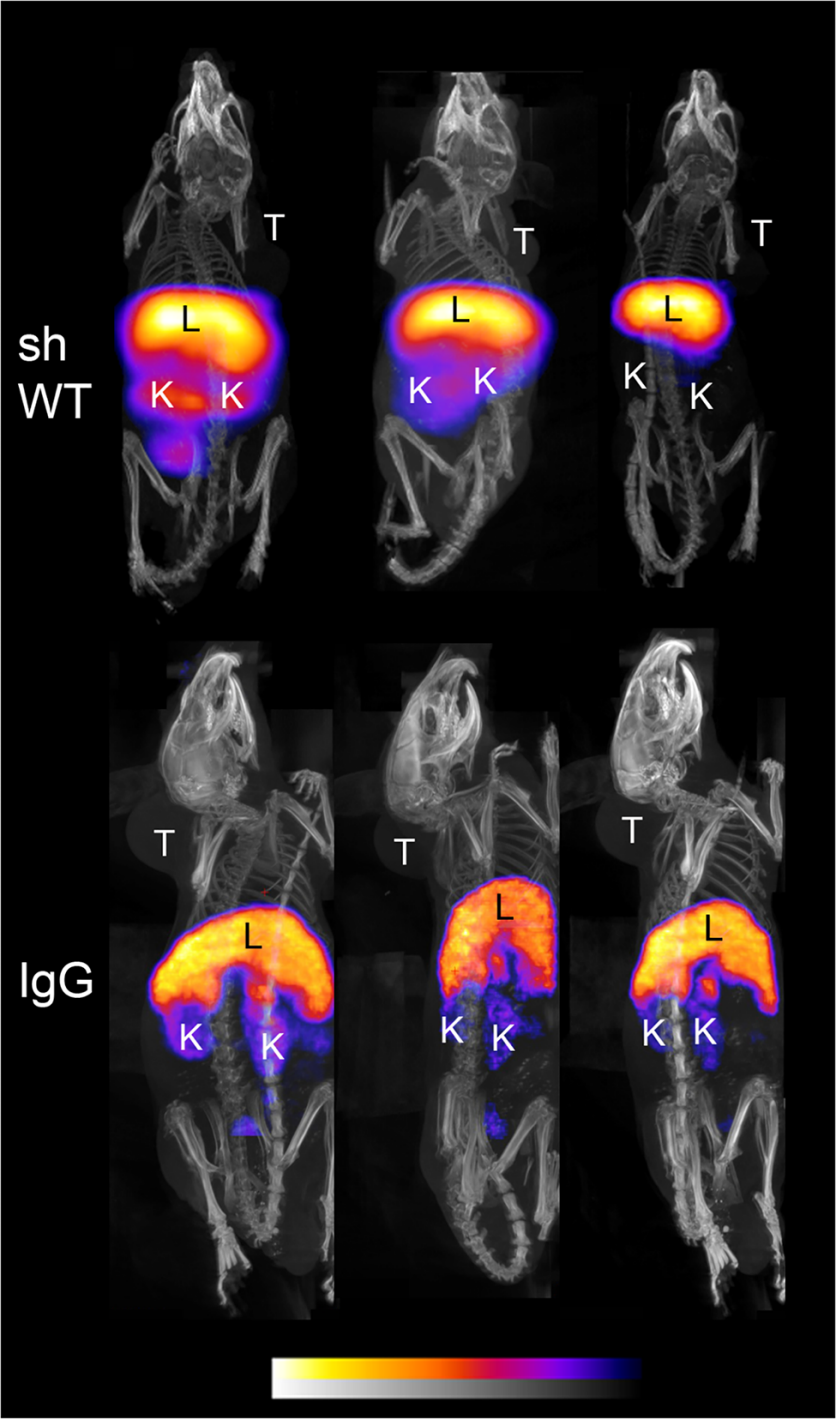
PET/CT imaging of mice bearing PC-3-tumors. Animals were imaged at 4 hours, 24 hours and 48 hours after injection of the ^64^Cu-MeCOSar radiotracer for shWTCH2 and IgG. Shown are the maximum-intensity projections. The color scale for the PET image data shows radiotracer uptake with white corresponding to the highest activity and blue to the lowest activity. T: tumor; K: kidney; L: liver.

The next generation ^64^Cu ligand 5-[(8-amino-3,6,10,13,16,19-hexaazabicyclo-[6.6.6]eico-1-yl)amino]-5-oxo-pentanoic acid hydrochloride (MeCOSar) was used and showed excellent and fast ^64^Cu binding at room temperature. Each animal received between 40–50 μg of radiolabeled Abdurin, corresponding to an activity of 4–7 MBq. Small-animal PET imaging and Standard Uptake Value (SUV) analysis of tumor bearing mice revealed an increasing tumor-uptake from 4.11 ± 0.77 at 4 hours, 9.22 ± 0.70 at 24 hours, to 10.89 ± 0.63 at 48 hours after tracer injection for ^64^CuMeCOSar-B11, and an accumulation of 3.95 ± 0.99 at 4 hours, 5.17 ± 1.33 at 24 hours, and 4.42 ± 0.89 at 48 hours after tracer injection for ^64^CuMeCOSar-B6 (Table 9, Fig 8). In contrast, tumor-bearing mice injected with the negative control ^64^CuMeCOSar-shWTCH2 showed only minor tracer uptake in their tumors, similar to background activity, at any measured time point and a similar lack of tumor uptake was seen for the positive control IgG, 64CUMeCOSar-IgG, which binds to EphA2 (Table 9, Fig 9). The ^64^CuMeCOSar-B11 Abdurin accumulated in the tumor faster and with a stronger SUV compared to the ^64^CuMeCOSar-B6 Abdurin. Non-tumor bearing mice showed the expected tissue enrichment in kidney and liver (Table 10).

**Table 9:** SUV of radiolabeled constructs in the tissues of mice bearing PC-3 tumors at 4 h, 24 h and 48 h after tracer injection. Results are presented as mean (+/-SD).

	**SUV of** ^**64**^ **CuMeCOSar-B6 (n = 3)**			**SUV of** ^**64**^ **CuMeCOSar-B11 (n = 3)**		
**Time**	**4 h**	**24 h**	**48 h**	**4 h**	**24 h**	**48 h**
**Tumor**	3.95±0.99	5.17±1.33	4.42±0.89	4.11±0.77	9.22±0.70	10.89±0.63
**Liver**	25.28±6.27	18.79±6.40	12.27±1.61	3.89±1.13	10.96±3.84	10.73±0.51
**Muscle**	0.29±0.11	0.21±0.05	0.17±0.05	0.36±0.26	0.42±0.49	0.39±0.05
	**SUV of** ^**64**^ **CuMeCOSar-shWTCH2 (n = 3)**			**SUV of** ^**64**^ **CuMeCOSar-IgG (n = 3)**		
**Time**	**4 h**	**24 h**	**48 h**	**4 h**	**24 h**	**48 h**
**Tumor**	4.02±0.17	3.55±0.16	3.40±0.87	0.37±0.14	0.93±0.30	1.01±0.11
**Liver**	3.23±0.36	2.46±0.58	1.37±0.84	8.36±3.22	9.82±3.70	7.97±3.43
**Muscle**	0.26±0.40	0.19±0.25	0.20±0.23	0.01±0.00	0.11±0.13	0.16±0.23

**Table 10:** SUV of radiolabeled constructs in the tissues of healthy mice at 4 h, 24 h and 48 h after tracer injection. Results are presented as mean (+/-SD).

	**SUV of** ^**64**^ **CuMeCOSar-B6 (n = 3)**			**SUV of** ^**64**^ **CuMeCOSar-B11 (n = 3)**		
**Time**	**4 h**	**24 h**	**48 h**	**4 h**	**24 h**	**48 h**
**Kidney**	33.21±12.59	39.31±16.40	26.40±14.34	39.31±16.40	26.98±12.88	20.82±5.82
**Liver**	31.63±17.95	33.64±5.83	28.90±18.64	33.64±5.83	25.90±7.78	20.86±3.24
**Muscle**	0.47±0.18	0.44±0.17	0.40±0.19	0.44±0.17	0.36±0.14	0.49±0.11
	**SUV of** ^**64**^ **CuMeCOSar-shWTCH2 (n = 3)**			**SUV of** ^**64**^ **CuMeCOSar-IgG (n = 3)**		
**Time**	**4 h**	**24 h**	**48 h**	**4 h**	**24 h**	**48 h**
**Kidney**	12.26±11.52	30.58±6.37	5.88±1.17	8.76±3.75	5.15±5.15	4.46±2.76
**Liver**	14.03±13.40	33.53±7.78	6.64±1.37	10.23±2.98	7.68±7.68	4.13±2.05
**Muscle**	0.08±0.08	0.30±0.11	0.08±0.01	0.11±0.16	0.05±0.05	0.09±0.07

### Biodistribution

**Fig 10:**
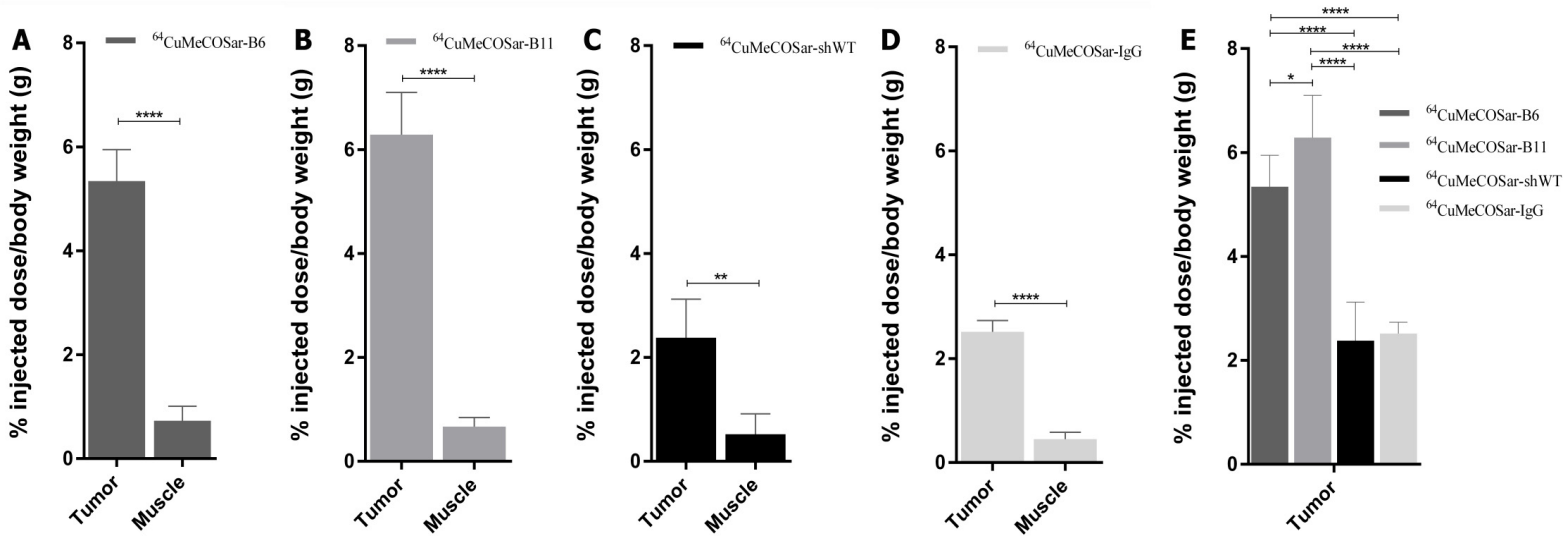
Biodistribution of Abdurins. The tissue uptake of: A) ^64^CuMeCOSar-B6 (n = 3)_,_ B) ^64^CuMeCOSar-B11 (n = 4) and C) control ^64^CuMeCOSar-shWTCH2 (n = 3); and D) ^64^CuMeCOSar-IgG (n = 3) at 48 hours after tracer injection. E) Direct comparison of tumor uptake between the three different constructs. Data are given as % injected dose per body weight (g) 48 hour after tracer injection (*/p = 0.01, **/p = 0.005, ***/p = 0.001, ****/p = 0.0001).

The *in vivo* biodistribution of ^64^Cu-MeCOSar–radiolabeled Abdurins was quantitatively assessed by γ-counting of selected isolated organs from tumor bearing mice 48 hours after tracer injection. The data indicated a significantly higher tumor uptake of B11 (6.29 ± 0.81%ID/g) and B6 (5.34 ± 0.60%ID/g), compared with the negative control shWTCH2 (2.38 ± 0.74%ID/g) and the positive control IgG (2.41+0.77%ID/g). Other organs with significant tracer uptake were the liver (9.38 ± 5.58%ID/g for the B11, 16.68 ± 3.16%ID/g for B6 and 9.32 ± 3.68%ID/g for shWTCH2) and kidney (17.29 ± 0.46%ID/g for the B11, 17.85 ± 2.94%ID/g for B6 and 12.12 ± 2.77%ID/g for shWTCH2). No significant radioactivity was detected in the muscle (0.67 ± 0.18%ID/g for the B11, 0.73 ± 0.28%ID/g for B6 and 0.52 ± 0.39%ID/g or for shWTCH2) (Fig 10). The high kidney activity indicates renal clearance in accordance with the size of the radiometal-labeled Abdurins. These results were confirmed in healthy mice.

## Discussion

The purpose of this study was to demonstrate that large, diverse, Abdurin DNA libraries can be constructed, formatted and screened using phage and DNA display, leading to the isolation of high affinity binders to targets such as the human tyrosine kinase receptor EphA2. Various libraries were constructed focusing on the three loop regions normally involved in Fc-gamma receptor binding of the CH2 domain. When screened on immobilized mEphA2, the initial clones isolated were all from the loop DE library. This was an unexpected result as all loops were previously engineered either by grafting amino acid sequences or using mutational library approaches. Loops BC and FG have been mutated to generate a combinatorial library comprising 10 amino acids in the BC loop and 6 amino acids in the FG loop. This library design resulted in specific binders to CD4 using phage display [30]. However, this work demonstrated that loop BC determined the binding specificity to CD4 and the loop BC sequence could not be grafted elsewhere within the framework and still bind CD4, suggesting that affinity for the antigen was dictated by the projection of the loop from the position of strands B and C. In another study, CDR-H3 sequences were grafted into loop FG of CH2D and then loop BC and DE were mutated to yield binders against the HIV-1 MPER peptide [31]. Although loop FG has conserved contacts with FcγRIIIa receptor [32], the results from the preceding studies anticipated that the loop FG library used to find binders to EphA2 would produce the initial binding clones, yet initial binders from library #16 were not observed. Interestingly, the libraries were designed to include functional amino acids such as Leucine and Proline. However, since binders were not isolated from library # 16, which would be expected to be enriched at conserved positions Leu328 and Pro329, it is postulated that the EphA2 antigen did not present an appropriate complementary epitope for the #16 FG loop library used in our study. The loop DE variants isolated from the primary selection were further tested using surface Plasmon resonance and ELISA on both human and mouse EphA2 expressing cells. Two dominant loop DE sequences emerged that bound equally well to murine and human EphA2, but only in the low micromolar to high nanomolar range. These loop DE variants were used to construct loop BC libraries for affinity maturation and screened using the CIS display method. After several rounds of screening, a single loop DE variant was retained and paired with several loop BC variants. Only loop BC variants from the longer Val266 to Pro271 library were isolated, possibly because additional randomization allowed greater opportunity to sample chemical or conformational compatibility with EphA2. Indeed, the sequences of the loop BC variants that bound EphA2 demonstrate conservation at the extremities of the loop: Tyrosine and Leucine being conserved in clones D2 and B11 at positions 266 and 271, respectively; Proline and Aspartic acid being conserved in clones G7 and B6 at positions 266 and 271, respectively. The new dual loop binders tested had low nM target binding by ELISA and surface Plasmon resonance assays, and in cell binding assays on hEphA2 expressing cells. In addition, all isolated binders had comparable affinities to both human and murine EphA2. This work highlights the benefit of using both phage and cell-free CIS display to rapidly isolate and optimize Abdurin binders to a target of interest. In addition, isolating binders that recognize both murine and human EphA2 can be helpful for nonclinical development and animal toxicology testing.

Going beyond cell-based assays, we wanted to determine if the EphA2-binding Abdurins could localize specifically into implanted PC-3 tumors using PET imaging and demonstrate that the localization and accumulation was specific and comparable to a full-length IgG, potentially demonstrating an advantage of greater tumor penetration due to the smaller size of the Abdurins. Radiolabeling of isolated Abdurins with ^64^Cu through a MeCOSar ligand [33] was accomplished without loss of target or FcRn binding. Small-animal PET imaging studies showed that the ^64^Cu-labeled Abdurins B6 and B11 specifically accumulated into implanted PC-3 tumors, whereas tracer uptake of the control Abdurin and the positive control IgG remained at background levels. Impressive tumor uptake in PC-3-positive tumors was already reached 24 hours after tracer injection for B6 and B11. The quality of the images improved further when the imaging was extended to 48 hours after injection. ^64^Cu–MeCOSar complexes are extremely stable *in vivo* [33, 34] and the present approach is ideally suited for long circulating antibody fragments such as the Abdurins. The rapid internalization of the Abdurins favors the metabolism and trapping of the radioactivity at the tumor site, reaching and maintaining a strong signal over time.

B6 and B11 bound and internalized into PC-3 cells equally well, and both Abdurins have similar affinities to human and murine EphA2. However, B11 accumulated into tumors to a significantly higher extent than did B6. The reasons for this difference are not known, but could be related to a number of factors including; a difference in the location of the ^64^Cu labeling, differences in serum stability, binding to an alternative epitope on EphA2, or the observation that B6 bound and accumulated to higher extent in the liver, thereby, sequestering B6 and preventing it from recirculating to the tumor site. In addition, B6 had a slightly higher affinity to murine EphA2 compared to B11, while B11 had a slightly higher affinity for human EphA2 compared to B6. Therefore, possibly explaining the higher accumulation of B6 in the liver. Further studies to understand the differences between the Abdurin binders are ongoing.

All the proteins tested localized in the liver. This result is not unexpected as the liver is a primary site for IgG and protein clearance. The higher concentration of the mAb, B6 and B11 Abdurin binders in the liver could be due, in part, to the expression of EphA2 on mouse hepatocytes and the ability of the antibody, B6 and B11 to bind murine EphA2 [19, 20]. In addition, localization in the liver is expected as the Abdurin binders also bind to FcRn receptors on liver endothelial cells. Therefore, the concentration of Abdurins, or the native CH2 domain, is likely to be higher in the liver compared to other organs. With regards to the observed kidney accumulation of Abdurins, the small size of the Abdurins places them under the molecular weight threshold for renal clearance and these results were also expected. The lack of tissue accumulation in other tissues supports the specificity of B6 and B11 targeting and a lack of EphA2 expression in the other normal tissues in the mouse.

The monoclonal antibody control used in these studies bound PC-3 cells with high affinity, readily internalized and was specific for the EphA2 receptor. However, using the Standard Uptake Values obtained from the PET images or using direct tissue quantification of the gamma counts from isolated organs, only 1–2% of the injected dose per gram of tumor tissue of the monoclonal antibody accumulated into the tumor at the 48 hour time point. It has been reported that smaller protein scaffolds penetrate and accumulate in tumors to a higher degree than larger proteins such as monoclonal antibodies [3, 9, 10, 34]. For many antibodies and antibody-drug conjugates (ADCs), it has been reported that the percentage of injected dose (ID) per gram of tumor tissue ranges from 0.08 to 0.003 in humans [35, 36, 37]. Based on the data reported herein for the Abdurin scaffold with its smaller size and longer serum half-life, we observed a five to ten fold increase in the amount of the injected dose of the Abdurin over the positive control monoclonal antibody. Adjusting the data by molecular weight, concentration and the number of chelated isotopes per protein molecule (Abdurins were 1:1 and IgG was 3:1 for the chelator:protein ratio), it is estimated that up to 300 fold more Abdurin molecules may have accumulated into the implanted tumors compared to full-length IgG in this animal model at 48 hours.

We propose that the reason for this difference in tumor accumulation between the Abdurins and the control IgG is related to the smaller size of the Abdurins coupled with a longer serum half-life. The Abdurin is approximately one tenth the molecular weight of the full IgG and should better penetrate and distribute into the tumor tissue compared to IgG. The improved tumor penetration may likely benefit from the prolonged serum half-life of the Abdurins as well. We expected reasonable IgG accumulation over the time course of this experiment (48 hours) as others have observed detectable values for IgG at 8 hours [37], however, in this present study the anti-EphA2 IgG accumulation was similar to background at 1–2% of the injected dose at 48 hours. It is possible that extending the experiment to seven days would result in a higher percentage of the injected dose of the antibody in the tumor due to the longer circulating half-life of antibodies. Tumor penetration of large protein molecules is influenced by several factors including: half-life of the molecule, molecular weight, and binding affinity [9, 10, 12, 36, 37]. In addition, target biology such as target concentration, internalization rate, target turn-over, target dimerization and signaling pathways can influence tumor penetration and distribution of antibodies and protein scaffolds [9, 10, 12, 14]. The Abdurins benefit by both being small and having an extended serum half-life through retained FcRn binding. It has also been reported that the high affinity of full-length antibodies results in an accumulation of the antibody at the edge of the tumor (the “binding site barrier” effect) which, potentially, can also limit tumor distribution [35, 36, 37]. With the reported poor tumor penetration of IgG and ADCs, Abdurin-drug or Abdurin-toxin conjugates may be used to deliver higher concentrations of a specific payload into tumor tissue, possibly resulting in an improved clinical benefit for patients. Such Abdurin-drug and Abdurin-toxin constructs are currently in development for future testing.

## Conclusions

The present study demonstrates that the new class of small, prolonged circulating Abdurins is an ideal tool for the development of novel PET tracers for cancer imaging and potentially for cancer therapy when conjugated with radioisotopes, small molecule drugs or as immunotoxins.
